# The association of weather conditions with day-to-day variability in physical activity in patients with COPD

**DOI:** 10.1183/23120541.00314-2023

**Published:** 2023-11-13

**Authors:** Astrid Blondeel, Fien Hermans, Sofie Breuls, Marieke Wuyts, Nikolaas De Maeyer, Thessa Verniest, Eric Derom, Ben Van Calster, Wim Janssens, Thierry Troosters, Heleen Demeyer

**Affiliations:** 1 Department of Rehabilitation Sciences, KU Leuven, Leuven, Belgium; 2 Department of Rehabilitation Sciences, Ghent University, Ghent, Belgium; 3 Clinical Department of Respiratory Diseases, Regional Hospital Heilig Hart Leuven, Leuven, Belgium; 4 Clinical Department of Respiratory Medicine, University Hospital Ghent, Ghent, Belgium; 5 Department of Development and Regeneration, KU Leuven, Leuven, Belgium; 6 Department of Chronic Diseases, Metabolism and Aging (CHROMETA) – BREATHE, KU Leuven, Leuven, Belgium; 7 Clinical Department of Respiratory Diseases, University Hospitals Leuven, Leuven, Belgium; 8 These authors contributed equally

## Abstract

**Background:**

While patients with COPD often cite weather conditions as a reason for inactivity, little is known about the relationship between physical activity (PA) and weather conditions. The present study investigated the association of day-to-day weather changes on PA in patients with COPD and investigated patient characteristics related to being more or less influenced by weather conditions.

**Methods:**

In this longitudinal analysis, device-based day-by-day step counts were objectively measured in COPD patients for up to 12 months. Daily meteorological data (temperature, precipitation, wind speed, hours of sunlight and daylight) were linked to the daily step count and individual and multivariable relationships were investigated using mixed-model effects. Individual R^2^ was calculated for every subject to investigate the estimated influence of weather conditions on a patient level and its relationship with patient characteristics.

**Results:**

We included 50 patients with a mean±sd follow-up time of 282±93 days, totalling 14 117 patient-days. Daily temperature showed a positive linear pattern up until an inflexion point, after which a negative association with increasing temperature was observed (p<0.0001). Sunshine and daylight time had a positive association with PA (p<0.0001). Precipitation and wind speed were negatively associated with PA (p<0.0001). The median per-patient R^2^ for overall weather conditions was 0.08, ranging from 0.00 to 0.42. No strong associations between patient characteristics and per-patient R^2^ were observed.

**Conclusion:**

Weather conditions are partly associated with PA in patients with COPD, yet the overall explained variance of PA due to weather conditions is rather low and varied strongly between individuals.

## Tweetable abstract

**Weather conditions are partly associated with objectively measured physical activity in patients with COPD, yet the overall explained variance of physical activity due to the weather is rather low and varies strongly between individuals** https://bit.ly/450Z5Vb

## Introduction

Physical activity (PA), defined as any bodily movement produced by skeletal muscles resulting in energy expenditure, is an important health-enhancing behaviour in healthy as well as in chronic diseased populations, as it is related to important health outcomes such as the occurrence of comorbidities or all-cause mortality [1]. Due to the unpleasant sensation of dyspnoea or leg fatigue during activities of daily life, patients with COPD are less active compared to healthy peers [2, 3]. A thorough understanding of PA is crucial to set up strategies to improve PA.

PA is a complex behaviour, determined by multiple aspects. Bauman *et al*. [4] showed that PA could be embedded within an ecological model, with several aspects affecting PA, such as personal, interpersonal and environmental factors, including the weather. The latter is often cited by patients with COPD as one of the perceived barriers to being physically active [5]. Indeed, several studies confirmed a higher activity level in summer compared to winter [6, 7]. However, the impact of detailed weather conditions on long-term series of day-by-day changes in daily life activities is less known. Only one longitudinal study from London (UK), investigating PA in 73 patients using a waist-worn pedometer, showed that patients were less active on rainy, overcast or colder days [8]. More in-depth insights are needed to support these prior findings.

If weather determines variability in PA, it is important to take this into account when including PA outcomes in research. A simple way to correct for weather in statistical analysis is to use season or to introduce the length of the days as a continuous covariate [9]. However, this covers the concept of weather only partly. Using more detailed weather information might improve the statistical analysis. In addition, identifying the importance of influencing weather conditions on PA plays a role in optimising interventions such as PA coaching interventions that aim to improve PA by providing self-monitoring and feedback. It may offer the opportunity to provide more individualised PA interventions with targeted feedback incorporating weather conditions. We could suspect that some patients are more prone to the influence of weather conditions than others, for example in patients who are less limited by disease symptoms, weather could be a more influencing factor compared to patients with limited capacity. Identifying these patients will allow us to individually discuss barriers of PA in a more objective manner.

Therefore, in the current study, we investigated whether granular weather conditions are associated with PA in patients with COPD. We hypothesised that, on top of daylight, day-by-day information on precipitation, sunshine, temperature and wind speed are associated with PA, expressed as steps per day. Second, we aimed to investigate the individual explained variance for the multivariable weather model and distinguish patient characteristics related to a higher explained variance by weather conditions.

## Methods

### Study population

For the present analysis, data of patients with COPD included in the sham group of an ongoing randomised controlled trial testing the effectiveness of a long-term PA coaching intervention (www.clinicaltrials.gov identifier NCT04139200) were included. Patients were recruited at the University Hospitals Leuven and Regional Hospital HH Leuven (Leuven, Belgium) between January 2020 and May 2022. Patients with a diagnosis of COPD (confirmed by spirometry (forced expiratory volume in 1 s (FEV_1_)/forced vital capacity (FVC) ≤0.70)), aged >40 years, a smoking history of ≥10 pack-years and no moderate or severe exacerbations within 4 weeks prior to inclusion were eligible for participation in the study. Patients actively involved in multidisciplinary pulmonary rehabilitation, on the waiting list for lung transplantation, unable to learn to work with a smartphone device or unable to improve PA due to musculoskeletal comorbidities were excluded. The study was approved by the ethical committee of University Hospitals Leuven (s62907) and all subjects signed the informed consent prior to data collection.

### Data collection

#### Physical activity

Patients were instructed to wear a wrist- or waist-worn activity tracker (Fitbit, San Francisco, CA, USA) on a daily basis during waking hours for 12 months. This step counter is valid to identify day-by-day changes in patients with COPD [10]. Data on daily step count were collected through the coaching smartphone application (m-PAC, AppsOnly). Step count data of patients recruited >3 months prior to data lock were used (data lock for this analysis: 21 May 2022). As data quality control, in the absence of information on wearing time, nonwearing days were defined as none or <70 recorded steps, which were excluded.

#### Meteorological data

Hourly data on meteorological parameters (*i.e.* precipitation, sunshine, wind speed and temperature) were provided by the Royal Meteorological Institute of Belgium (KMI) from the nearest weather station to the hospital (*i.e.* Ukkel (precipitation and sunshine) and Zaventem (temperature and wind speed)). Only weather data between 07:00 and 22:00 were retained for further analyses. The following variables were calculated per day: minimum temperature (°C), maximum temperature (°C), mean temperature (°C), total amount of precipitation (mm), hours of precipitation (defined as ≥0.1 mm rainfall), total amount of sunshine (hours), maximal wind speed (m·s^−1^) and mean wind speed (m·s^−1^). The amount of daylight (min) was calculated based on the latitude and day of the year and was not restricted between 07:00 and 22:00 [9]. Days were classified as rainy and nonrainy days, in which a rainy day was defined as ≥0.1 mm rainfall between 07:00 and 22:00.

#### Patient characterisation

At baseline, patients performed the following clinical assessments: 1) a post-bronchodilator spirometry (according to the American Thoracic Society (ATS)/European Respiratory Society (ERS) guidelines) retrieving FEV_1_ and FVC [11]; 2) functional exercise capacity measured by the best out of two 6-min walk tests conducted in a 50-m corridor using standardised encouragement following ATS/ERS recommendations [12]; 3) symptoms of dyspnoea assessed by the modified Medical Research Council dyspnoea scale [13]; and 4) health status investigated using the COPD Assessment Test [14].

### Statistical analysis

Strong multicollinearity between similar weather variables was expected. Based on pairwise Spearman correlations (supplementary table S1), we retained daylight time, rainy days, hours of precipitation, mean temperature, mean wind speed and hours of sunshine as variables for the multivariable mixed-model analyses. The mixed models include a random effect for subject. The baseline model included time in study and weekend *versus* weekdays. Then, we added individual weather variables to this baseline model. Finally, we added all weather variables together. Model diagnostics were investigated and showed a limited skewness in the normal distribution of the residuals. The shape of the associations between continuous weather variables and PA were inspected visually using univariable models in which the weather variable was modelled using smoothing splines (supplementary figure S1). In case the association was considered nonlinear (*i.e.* for temperature and sunshine), variables were categorised.

For the second research question, first, individual R^2^ was calculated for every subject only using the baseline model, including time in study and weekend *versus* weekday variables. Next, individual R^2^ was calculated for every subject based on the multivariable model with daylight and weather variables included. To investigate the explained variance of the weather variable of the multivariable model on a patient level, the increase in individual R^2^ between the first and the second model was calculated for every subject. The relationship between patient characteristics and increase in individual R^2^ was investigated using Spearman correlation. All statistical analyses were performed using the SAS statistical package (V9.4; SAS Institute, Cary, NC, USA). Baseline data are presented as mean±sd and range; individual and multivariable associations are presented as coefficient (β) estimates, standard errors and 95% confidence intervals. Categorical variables are presented as percentages. Statistical significance was set at p<0.05 for all analyses.

## Results

### Baseline characteristics

**FIGURE 1:**
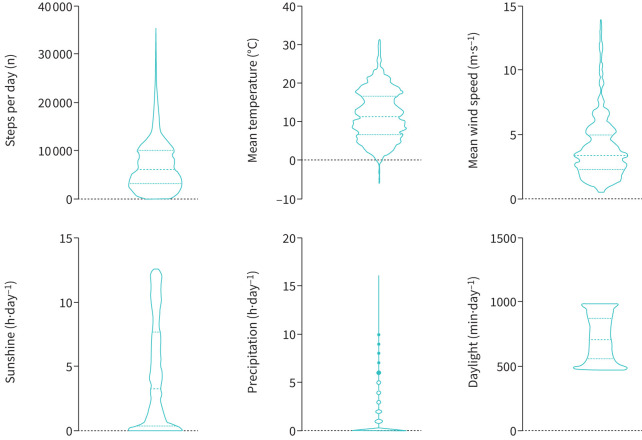
Distribution of physical activity (steps per day) and meteorological data in our sample: mean temperature (°C), mean wind speed (m·s^−1^), sunshine (h·day^−1^), hours of rain per day and daylight time (min·day^−1^).

58 patients were randomised to the sham group at the moment of database lock. Eight patients were not retained in the further analysis, as these patients were not yet 3 months into the study at the moment of database lock (n=7) or dropped out of the study after 1 week due to lower back pain (n=1). In the present study, PA data of 50 patients with COPD were combined with meteorological data. Patients included in the analyses were aged a mean±sd 66±7 years, with an average airflow obstruction of 56±17% predicted. More details on patient and weather characteristics are shown in table 1. The distribution of PA and meteorological data of our sample collected in Belgium is graphically represented in figure 1. A mean±sd (range) follow-up time of 282±93 (86–366) days per patient were available, resulting in a total of 14 117 patient-days. We excluded 711 (5%) patient-days with <70 steps per day recorded. No differences in weather conditions were observed between the days with step count below or above 70 steps per day (supplementary table S2). The majority of the patients used a wrist-worn activity tracker (n=40); 10 patients (20%) used a waist-worn consumer-based activity tracker.

**TABLE 1:** Patient characteristics (n=50) and average weather characteristics

**Patient characteristics**	
Age (years)	66±7 (45–82)
Male	62%
FEV_1_ (% pred)	56±17 (27–94)
6MWD (m)	478±83 (237–612)
BMI (kg·m^−2^)	26±4 (15–35)
mMRC score	1.7±0.9 (0–4)
CAT score	18±7 (2–33)
Mean steps per day^#^ (n)	7275±5311 (70–35 373)
Standard deviation of individual step count (n·day^−1^)	2516±1243 (707–6671)
Distance to weather station Zaventem (km)	22±13 (4.6–60.9)
Distance to weather station Ukkel (km)	31±14 (9.1–70.3)
**Weather characteristics^¶^**	
Mean temperature (°C)	12±6 (−6–31)
Minimum temperature (°C)	8±6 (−9–25)
Maximum temperature (°C)	14±7 (−5–36)
Precipitation (mm·day^−1^)	1.9±4.6 (0–54.7)
Precipitation (h·day^−1^)	2.0±3.2 (0–16)
Rainy days	45%
Sunshine (h·day^−1^)	4.3±4.1 (0–12.5)
Days with <5 h sunshine	61%
Days with 5**–**10 h sunshine	24%
Days with >10 h sunshine	15%
Daylight (min·day^−1^)	719±170 (477–991)
Mean wind speed (m·s^−1^)	3.8±2.0 (0.6–13.9)

Data are presented as mean±sd (range), unless otherwise stated. FEV_1_: forced expiratory volume in 1 s; 6MWD: 6-min walk distance; BMI: body mass index; mMRC: modified Medical Research Council dyspnoea score; CAT: COPD Assessment Test. ^#^: calculated based on the complete Fitbit dataset; ^¶^: weather conditions calculated based on all days with available corresponding physical activity data.

### Analysis of individual weather variables

**FIGURE 2:**
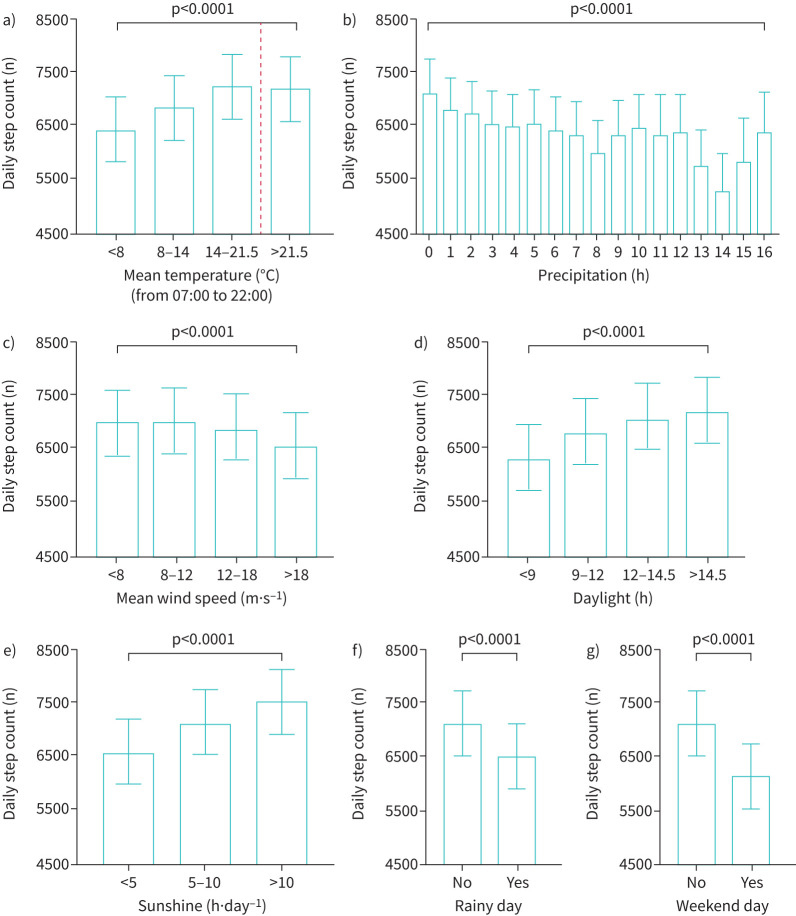
Graphical representation of individual association between weather conditions (collected between 07:00 and 22:00) and daily physical activity, expressed as marginal mean±se. a) Mean temperature as class variable, with temperature <21.5°C divided into tertiles. The inflection point (21.5°C) is indicated by the dashed line; b) hours of precipitation (*i.e.* number of hours with ≥0.1 mm rain); c) mean wind speed (m·s^−1^), divided into quartiles; d) hours of daylight, divided into quartiles; e) hours of sunshine, divided into meaningful subgroups of <5 h·day^−1^ sunshine *versus* 5–10 h·day^−1^ sunshine or >10 h·day^−1^ of sunshine; f) presence of a rainy day (*i.e.* ≥0.1 mm rain during the day); g) weekday *versus* weekend day.

In the baseline model with only days in study and weekend *versus* weekdays, both variables were clearly associated with step count. More days in study was associated with lower activity (β −1.3 steps per day; p<0.0001), and patients were less active on weekend days compared to weekdays (β −974 steps per day; p<0.0001). Associations of individual weather variables with daily step count, corrected for days in study and weekend *versus* weekdays, are shown in table 2 and figure 2. Daily mean, minimum and maximum temperature showed a positive linear pattern up until an inflexion point, whereafter a negative association with increasing temperature was observed (figure 2 and supplementary figure S1). To account for this nonlinear pattern, the individual association with temperature provided in table 2 was calculated for days with mean temperature <21.5°C. Based on the individual continuous analysis, with increasing temperature per centigrade (up until 21.5°C), an increase in parameter estimate±se of 57±4 steps per day was observed (p<0.0001). With further increasing temperature >21.5°C, a negative association was found. Hours of sunshine and daylight time showed a significant positive association with daily step count (p<0.0001). The total volume of precipitation as well as the number of hours with precipitation showed a significant linear negative association with daily steps (p<0.0001). On a rainy day, patients had a decrease in parameter estimate±se of 538±52 steps per day as compared to days without rain (p<0.0001). Mean and maximum wind speed were negatively associated with PA (respectively, parameter estimate±se: −96±12 steps per day and −67±9 steps per day; p<0.0001).

**TABLE 2:** Linear mixed-model analyses when adding individual weather conditions to the baseline model (time in study and weekdays *versus* weekend days); dependent variable: daily step count

	**Parameter estimate β±se**	**95% CI**	**p-value**
**Precipitation-related factors**			
Precipitation (mm·day^−1^)	−40.1±5.3	−50.4 to −29.8	<0.0001
Rainy day			
No (ref.)			
Yes	−625.2±48.8	−720.9 to −529.5	<0.0001
Precipitation (h·day^−1^)	−95.7±7.6	−110.6 to −80.8	<0.0001
**Wind-related factors**			
Maximum wind speed (m·s^−1^)	−66.9±9.4	−85.4 to −48.4	<0.0001
Mean wind speed (m·s^−1^)	−96.1±12.2	−120 to −72.1	<0.0001
**Temperature-related factors**			
Minimum temperature^#^ (°C)	40.5±4.7	31.4 to 49.7	<0.0001
Maximum temperature^#^ (°C)	58.0±4.0	50.1 to 65.9	<0.0001
Mean temperature^#^ (°C)	57.1±4.4	48.4 to 65.7	<0.0001
Mean temperature			
<8°C	−816.5±63.0	−940.0 to −693.1	<0.0001
8**–**14°C	−397.4±61.5	−518.1 to −276.8	<0.0001
14**–**21.5°C (ref.)			
>21.5°C	−56.7±118.5	−289.0 to 175.6	0.63
**Sun-related factors**			
Sunshine <5 h·day^−1^	−605.8±58.1	−719.6 to −491.9	<0.0001
5**–**10 h·day^−1^ (ref.)			
>10 h·day^−1^	342.7±79.4	187.2 to 498.3	<0.0001
Daylight (min·day^−1^)	1.9±0.1	1.6 to 2.1	<0.0001

Ref.: reference value. ^#^: estimates of temperature as continuous variable are presented for days with mean temperature <21.5°C.

### Multivariable analysis including all weather conditions

The multivariable model is shown in table 3. The model was corrected for time in study and weekend *versus* weekdays, of which estimates were comparable to individual analyses.

**TABLE 3:** Multivariable linear mixed-model analysis for weather conditions as independent variables and daily step count as dependent variable, corrected for time in study and weekend *versus* weekdays

	**Parameter estimate β±se**	**95% CI**	**p*-*value**
**Rainy day**			
No (ref.)			
Yes	−190.3±76.2	−339.7 to −40.9	0.01
**Precipitation (h·day^−1^**)	−31.7±11.0	−53.3 to −10.1	<0.01
**Mean wind speed (m·s^−1^)**	−20.4±13.8	−47.5 to 6.7	<0.01
**Mean temperature**			
<8°C	−372.3±86.3	−541.5 to −203.2	<0.0001
8**–**14°C	−121.6±72.3	−263.4 to 20.1	0.09
14**–**21.5°C (ref.)			
>21.5°C	−497.5±119.5	−731.7 to −263.3	<0.0001
**Sunshine**			
<5 h·day^−1^	−312.7±66.5	−443.0 to −182.4	<0.0001
5**–**10 h·day^−1^ (ref.)			
>10 h·day^−1^	229.5±81.8	69.2 to 389.8	<0.01
Daylight (min·day^−1^)	0.74±0.22	0.3 to 1.2	<0.01
**Time in study (days)**	−1.4±0.3	−1.9 to −0.9	<0.0001
**Weekend days (ref.: weekdays)**	−982.6±53.8	−1088 to −877	<0.0001

Ref.: reference value.

### Individual explained variance to weather conditions

**FIGURE 3:**
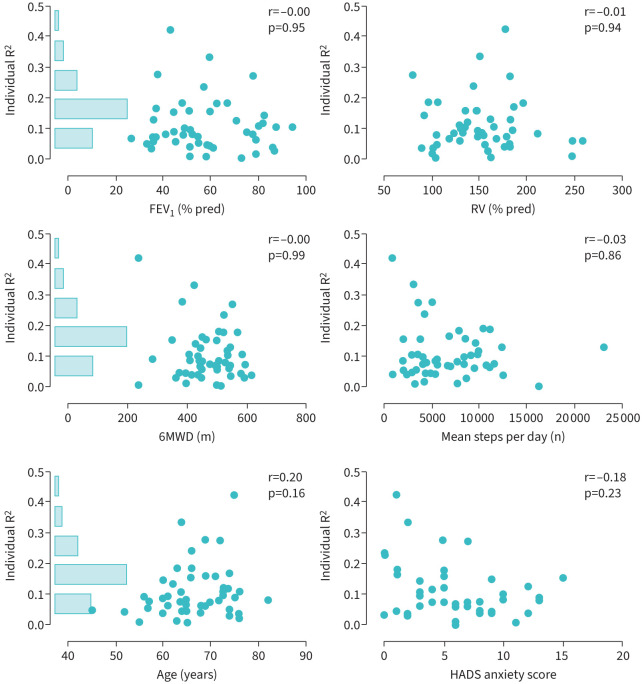
The relationship between individual R^2^ and airflow obstruction (forced expiratory volume in 1s (FEV_1_) % pred), hyperinflation (residual volume (RV) % pred), exercise capacity (6-min walk distance (6MWD)), physical activity (steps per day), age and anxiety (measured by Hospital Anxiety and Depression Scale (HADS) questionnaire). Bars indicate the frequency distribution of R^2^.

Calculating the individual R^2^ using only time in study and weekend *versus* weekdays in the model resulted in a median R^2^ of 0.07. By adding daylight and weather variables to the multivariable model, median R^2^ increased to 0.18, ranging from 0.03 to 0.72. The individual R^2^ accounting for weather-related factors (median 0.08, range 0.00–0.42) was not strongly related to anxiety score and age and not related to airflow obstruction (FEV_1_ % pred), hyperinflation (residual volume % pred), average physical activity level and exercise capacity (expressed as 6-min walk distance), with absolute Spearman correlations up to 0.20 (figure 3).

## Discussion

Our longitudinal study provides evidence that patients with COPD are more active on warmer and sunnier days, and less active on rainy days. Very warm days (*i.e.* mean temperature >21.5°C) were associated with a decrease of PA in patients with COPD. While these findings are intuitive, the present study provided in-depth insight into the magnitude of the association between weather and PA. To visualise the interpretation of the multivariable model, one can calculate the theoretical difference between worst possible weather condition and best possible weather condition using the estimates of the different variables. Between the worst possible weather condition (*i.e.* worst-case scenario: full day of rain with low daylight time, mean temperature <8°C, very windy and low amount of sunshine) and best possible weather condition (*i.e.* best-case scenario: no precipitation, long day with >10 h of sunshine, mean temperature between 14 and 21.5°C and low wind speed), an average range of 1985 steps per day was observed based on the multivariable model. This shows that weather accounts for an important but partial contribution of the within-subject variability in PA. Analyses on the individual association to weather conditions showed that this response varies among individuals and that the overall explained variance of PA due to weather conditions is rather small, with R^2^ ranging from 0.00 to 0.42. When aiming to identify differences in patient characteristics for subjects of which PA was more or less associated with weather, no strong correlations were found.

Our data indicate that, when evaluating PA or the effect of interventions on PA, weather should be taken into account [9, 15]. Previous research proposed that daylight time can be an accurate proxy to use as a covariate when evaluating PA over time [9, 15]. Based on the multivariable model, we assert that detailed information on different weather variables, on top of daylight, provide added value to describe variations in PA. However, to use all these weather variables in PA analyses, this might compromise the complexity towards study results. Nonetheless, integrating day-by-day information on temperature and precipitation might already improve the prediction of PA based on weather conditions.

Our findings are in line with previous research data from London [8], as they showed a positive association with temperature and step count up until an inflexion point, whereby a negative association with further increasing temperature was observed. The magnitude of the associations were smaller in comparison to the present study; however, patients in our study were overall more active compared to this study (on average 7275 steps per day *versus* 3767 steps per day, respectively), yet this might also be due the use and placement of the activity tracker (wrist- or waist-worn activity tracker *versus* wrist-worn pedometer, respectively). A large multicentre study in Europe only reported a negative association of rainfall on amount and intensity of PA [16]. In contrast to our data, these associations were based on the weekly average PA measurement and not on a day-to-day basis. In the current study, we are able to collect more detailed spectrum of weather conditions and PA. The aforementioned studies were based in Europe, with similar climate. However, based on research comparing the influence of weather in two different climates (*i.e.* Belgium and Brazil), we believe that these results can be generalised to other climates. The study by Furlanetto *et al*. [6], comparing the influence of weather in these two different climates, showed that patients were less active in winter as compared to summer, both in Belgium and Brazil, with comparable associations of mean temperature in their (+1 min of activity) and our (+60 steps) study.

Supporting our objective findings on the association between weather and PA, qualitative research suggested that poor weather conditions are the most frequent reported reason by patients for sedentary behaviour [5]. In the general population, subjects who were less likely to perceive the weather as a barrier were more likely to be high-volume walkers [17]. The association between weather conditions and respiratory symptoms or lung function could partly explain our observed findings. Previous literature suggested that both high and low environmental temperatures are associated with reduced lung function [18, 19]. Scheerens *et al*. [20] found an increase in breathing symptoms (*i.e.* breathlessness, wheezing, tightness of the chest) with higher temperatures and an increase in bronchitis symptoms (*i.e.* cough, sputum production) with colder temperatures. As we did not collect data on patient-reported symptoms, we cannot further elaborate on this with the current dataset.

The association between poor weather conditions and low activity levels suggests the need to take weather conditions into account when stimulating patients towards a more active lifestyle by anticipating towards strategies to overcome the burden of poor weather conditions. Our data suggest that this should probably be done on an individual level, as we see large variability in the association between weather conditions and day-to-day PA. Unfortunately, no relationships between per-patient R^2^ and patient characteristics such as lung function (airflow obstruction and hyperinflation), exercise capacity and physical activity level were observed. In contrast to our hypothesis, we were not able to identify patients who are more prone to be influenced by weather conditions. Therefore, possibly, the best way to tailor a PA coaching intervention is to interview patients on their perceived barriers for PA prior to starting a coaching intervention. Alternative strategies to be active even when the weather is bad, such as indoor activities or exercises, should be discussed and encouraged. Future research on PA-enhancing interventions might evaluate the effect of adding weather-tailored activity suggestions or feedback to patients.

### Strengths and limitations

This study uses a unique longitudinal dataset, containing device-based data of daily PA, objectively measured by a consumer-based activity tracker and hourly data on different weather conditions, in 50 patients with COPD with an average follow-up period of±9 months (range 3–12 months). Due to the large follow-up period, a broad spectrum of weather conditions was covered in our dataset. Individual and multivariable mixed-model analyses give us insights into the direction and magnitude of the associations. However, we cannot provide insights on the role of air pollution or respiratory symptoms. To the best of our knowledge, we were the first to evaluate the individual association to overall weather conditions. Contrary to our hypothesis, we failed to identify patient characteristics related to being strongly or limited influenced by the weather.

Some limitations should be taken into account. In the present analysis, categorisation of continuous variables (*i.e.* temperature and sunshine) were used to account for the nonlinear association. We acknowledge that from a statistical point of view this can be discouraged; however, meaningful cut-offs were used to ensure comprehensible interpretation of the data [21]. As the use of a consumer-based activity tracker to measure PA in daily life results in a large longitudinal dataset, an indication of wearing time was not available, which contrasts with the use of a validated accelerometer to measure PA in patients with COPD [22]. To take this into account, we excluded days with a step count of none or <70 steps for analyses, to exclude nonwearing days. We are aware that this does not fully control for days with only a limited amount of wearing time. In the present study, both wrist- and waist-worn activity trackers were used; however, this should not limit the interpretation of the current analyses, as we previously validated these wrist- and waist-worn consumer-based activity trackers as coaching tool and these devices were able to pick up day-by-day variability similar to a validated activity monitor (Dynaport MoveMonitor) [10]. Due to the use of a consumer-based activity tracker, we could only capture one dimension of PA (*i.e.* steps per day); no information on intensity of PA was provided. In the present study, a convenient sample of 50 patients was evaluated. Yet, this sample size might have been too small to explore patient characteristics related to individual associations with weather conditions. Finally, weather data were collected from the weather stations in Ukkel (for temperature, sun and rain) and Zaventem (for wind), which differ from the exact locations of the patients. However, the average distance from the weather station in Ukkel to the patient's home was limited (31±15 km). We expect that weather does not vary much within those locations, especially when aggregated over a day.

### Conclusion

Weather conditions are partly associated with PA in patients with COPD, as patients are less active on days with rain and more active on warmer and sunnier days. Analyses on the individual association to weather conditions showed that this response varies among individuals and that the overall explained variance of PA due to weather conditions is rather low. When providing interventions aiming to increase PA in patients with COPD, weather conditions should be taken into account.

## Supplementary material

10.1183/23120541.00314-2023.Supp1 **Please note:** supplementary material is not edited by the Editorial Office, and is uploaded as it has been supplied by the author.
Supplementary material 00314-2023.SUPPLEMENT
