# Supplementary material for: The association of weather conditions with day-to-day variability in physical activity in patients with COPD
Source: ERJ Open Res. 2023 Nov 13;9(6):00314-2023. doi: 10.1183/23120541.00314-2023 (PMC10641577; doi:10.1183/23120541.00314-2023)

The association of weather conditions with day-to-day variability in physical activity in patients with COPD

Astrid Blondeel, Fien Hermans′ Sofie Breuls, Marieke Wuyts, Nikolaas De Maeyer, Thessa Verniest, Eric Derom, Ben Van Calster, Wim Janssens, Thierry Troosters, Heleen Demeyer

Supplementary file

***Figure S1**: smoothed curves (splines) of daily step count for different weather conditions separately (adjusted for days in study).*

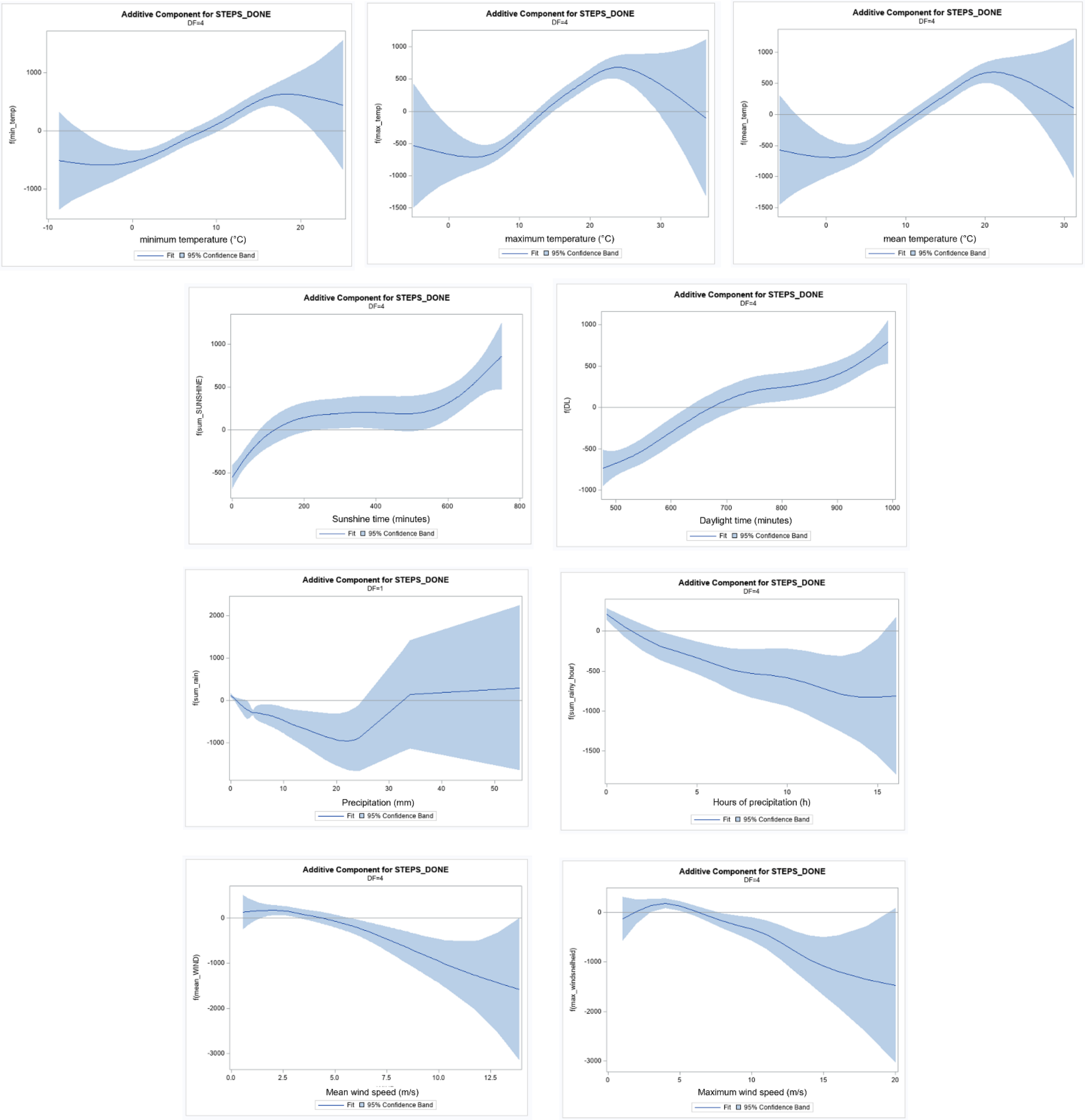

Table S1: *Spearman correlation matrix for different weather variables*

	**Mean temp**	**Max temp**	**Min temp**	**Mean wind**	**Max wind**	**Precipita-tion**	**Hours of precipita-tion**	**Sunshine**	**Daylight**
**Mean temp**	1,00	0,99<br><,0001	0,95<br><,0001	-0,10<br><,0001	-0,08<br><,0001	-0.10<br><,0001	-0,16<br><,0001	0,41<br><,0001	0,75<br><,0001
**Max temp**	0,99<br><,0001	1,00	0,91<br><,0001	-0,14<br><,0001	-0,11<br><,0001	-0.15<br>0,01	-0,21<br><,0001	0,50<br><,0001	0,77<br><,0001
**Min temp**	0,95<br><,0001	0,91<br><,0001	1,00	-0,01<br>0.21	-0,01<br>0,59	0,03<br><,0001	-0,01<br>0.11	0,21<br><,0001	0,68<br><,0001
**Mean wind**	-0,10<br><,0001	-0,14<br><,0001	-0,01<br><,0001	1,00	0,94<br><,0001	0,42<br><,0001	0,44<br><,0001	-0,26<br><,0001	-0,14<br><,0001
**Max wind**	-0,08<br><,0001	-0,11<br><,0001	-0,01<br>0,59	0,94<br><,0001	1,00	0,44<br><,0001	0,45<br><,0001	-0,23<br><,0001	-0,08<br><,0001
**Precipitation**	-0.10<br><,0001	-0.15<br><,0001	0,03<br><,0001	0,42<br><,0001	0,44<br><,0001	1,00	0,97<br><,0001	-0,54<br><,0001	-0,10<br><,0001
**Hours of precipitation**	-0,16<br><,0001	-0,22<br><,0001	-0,01<br>0.11	0,44<br><,0001	0,45<br><,0001	0,97<br><,0001	1,00	-0,59<br><,0001	-0,16<br><,0001
**Sunshine**	0,42<br><,0001	0,50<br><,0001	0,21<br><,0001	-0,26<br><,0001	-0,23<br><,0001	-0,55<br><,0001	-0,59<br><,0001	1,00	0,45<br><,0001
**Daylight**	0,75<br><,0001	0,77<br><,0001	0,68<br><,0001	-0,14<br><,0001	-0,08<br><,0001	-0.10<br><,0001	-0,16<br><,0001	0,45<br><,0001	1,00

Table S2: *weather conditions on days included in the analysis and days excluded due to non-wearing days (step count below 70 steps)*

**Weather characteristics**	**Wearing days (steps > 70)**	**Non wearing days (steps < 70)**
Mean temperature (°C)	12 ± 6	12 ± 6
Precipitation (mm/day)	1.9 ± 4.6	1.9 ± 4.2
Hours of precipitation per day (h/day)	2.0 ± 3.2	2.1 ± 3.4
Rainy day (%)	45 %	44 %
Sunshine (min/day)	261 ± 245	278 ± 247
Daylight (min/day)	719 ± 170	736 ± 159
Mean wind speed (m/s)	3.8 ± 2.0	3.9 ± 2.2

Table S3: *univariable linear mixed model analysis for weather conditions as independent variables and daily step count as dependent variable, not corrected for time in study and weekend versus weekdays.*

		Univariable association		
**Dependent variable: daily step count**		Estimate	SE	*P-value*
Precipitation (mm/day)		-46.0	5.3	<0.0001
Rainy day	no (ref)			
	yes	-605.5	49.4	<0.0001
Hours of precipitation per day		-96.3	7.7	<0.0001
Max wind speed (m/s)		-66.7	9.5	<0.0001
Mean wind speed (m/s)		-93.5	12.4	<0.0001
Minimum temperature (°C)		43.4	4.7	<0.0001
Maximum temperature (°C)		59.5	4.1	<0.0001
Mean temperature (°C)		59.4	4.4	<0.0001
Sunshine	<5h/day	-553.5	58.8	<0.0001
	5-10h/day (ref)			
	>10h/day	376.9	80.4	<0.0001
Daylight (min/day)		1.9	0.1	<0.0001

Figure S2: *graphical representation of individual association between mean temperature (as class variable, divided per 2.5°C) and daily PA, expressed as marginal means (SE). The inflection point (21.5°C) is indicated by the red dotted line.*

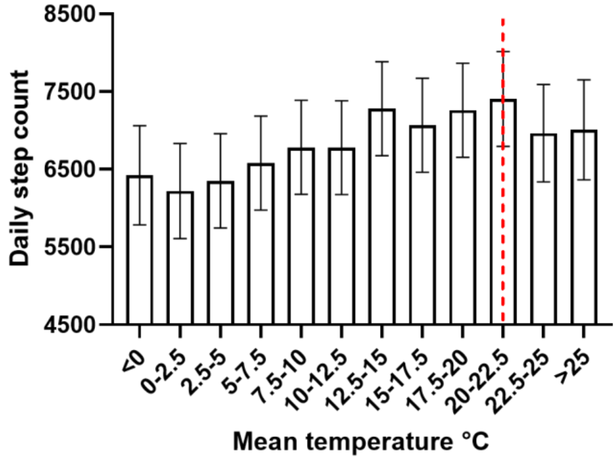

Supplement: Supplementary file 1 [file 00314-2023.SUPPLEMENT.pdf]
